# Nurses’ perspectives on workarounds in clinical practice: A phenomenological analysis

**DOI:** 10.1111/jocn.16110

**Published:** 2021-11-09

**Authors:** Monica Bianchi, Luca Ghirotto

**Affiliations:** ^1^ Department of Business Economics, Health and Social Care University of Applied Sciences and Arts of Southern Switzerland Manno Switzerland; ^2^ Azienda USL – IRCCS di Reggio Emilia Reggio Emilia Italy

**Keywords:** clinical placement, nurse, nurses’ responsibilities, phenomenology, protocol, safeguarding

## Abstract

**Aims and objectives:**

To explore the phenomenon of workarounds in clinical practice through the nurses’ perspective and identify which factors according to their experience contribute to activities carried forth non‐compliantly to procedures, protocols and rules defined by an Institution.

**Background:**

A workaround in clinical practice is a non‐compliance and a violation of an organisation's defined procedures, regulations or rules that may prevent adverse events. Its increasing recurrence in the workplace calls for a deeper analysis of the phenomenon.

**Design:**

A phenomenological descriptive design, following Colaizzi's analysis.

**Methods:**

In‐depth interviews were conducted with 16 nurses about their experience of workarounds. The interviews were digitally audio‐recorded and transcribed verbatim. Two researchers conducted data analysis independently and followed three phases: extracting significant statements, creating formulated meanings and aggregating them into themes. The process employed NVivo 12 software. COREQ checklist was used for reporting.

**Results:**

Data analysis identified 17 sub‐themes falling into the four macro‐themes: (i) living the profession in saved times; (ii) Perceiving contingencies as a guide to action; (iii) sense of personal responsibility; and (iv) emotional aspects.

**Conclusions:**

From a nurses’ perspective, a workaround is often triggered by the need to overcome problems interfering with efficient and timely patient care in everyday clinical practice. This will of undertaking responsibilities favouring gained efficiency is closely linked to their confidence acquired over years of experience in the field.

**Relevance to clinical practice:**

The results of this study can help clinical leadership to acknowledge workaround, understand the underlying triggers and work towards reconciling official procedures with real‐world situations. They can help nurses working in clinical practice to reflect and understand how to reconcile the needs related to the demands of organisations with the need to live their profession, which is more patient‐oriented.

**What does this paper contribute to the wider global community?**

- Findings have evidenced workaround as a symptom of unmet professional needs and lack of time or tools to complete their tasks by the book.
- Workaround are linked to the professional needs: the most important is to have time to express the fundamental meaning of being a nurse.
- An organizational culture where nurses can share responsibilities, have support from the leaders, be empowered, and be involved in the changes contribute to reducing workaround.

## 1 INTRODUCTION

In the health field, both the standardisation of behaviours and creativity and imagination are necessary to overcome and solve the problems that can arise every day (Debono et al., 2013; Reason, 2010). In general, to improve the safety level of the activities carried out, standardised procedures are defined for carrying out activities considered critical (Laverty et al., 2015; Shekelle et al., 2013). When these strategies are adopted, it prevails in the organisations reading the human element as a source of fragility in the performance of nursing processes (Reason, 2010). Despite this, it is the same organisations that require the staff to keep high flexibility in behaviour to allow responsiveness to continuous adjustments of operational processes linked to changes in technology, users and organisation requests; to extraordinary events that must be managed in an unexpected and ‘original’ way; the need to use the individual and collective intelligence of professionals to improve the existing situation. In all these cases, the use of creativity by professionals is judged positively (Debono et al., 2013; Reason, 2010). It starts from these two contradictory situations that we observe behaviours acted by the staff, sometimes in a rigid and pre‐defined way, other times creatively. Analysing these behaviours, we observe the presence of phenomena such as violations of defined rules (e.g. in protocols or instructions that have been designed to act with greater safety) (Reason, 2010) and workarounds taken by professionals to try to overcome obstacles or unforeseen events in clinical practice (Debono et al., 2013). Related to the creativity of using a workaround in nursing practice, Vestal (2008) reports that nurses use it because they feel responsible for doing everything well. Nurses may turn the art of workaround into a new way of work experience, deeming some workarounds as more creatives than prescribed solutions. In daily practice, workarounds should substitute the official process that is not working well or avoid unrealistic or harmful demands (Vestal, 2008).

First definitions of workarounds in health care (Halbesleben et al., 2008; Koppel et al., 2008) are related to actions taken by a professional or a group to obtain a work goal. In this context, this process is hindered by time‐consuming activities or events that nurses consider mistake‐proofing. The international scientific debate describes how the professionals use workarounds to solve limitations in procedures or when they are not familiar with them, to keep away from standards, achieve different goals, save time or achieve other objectives in the healthcare system (Tucker et al., 2020). Then, workarounds are described both as irrelevant and essential to the conduct of daily work as both objectionable, undesirable, dangerous and even as unethical or illegal violations of procedures and responsibilities (Alter, 2014).

In the past decade, there has been a growing narrative portraying nurses as masters of workarounds, that is in the practice of finding alternative solutions for bypassing the stiffness of regulations, procedures or protocols (Dierynck et al., 2017) difficulty adaptable to sudden changes in patients’ conditions. Protocols hinder quick decision‐making and efficiency (Halbesleben et al., 2013). Although workarounds have coexisted alongside official clinical practice, the ever more cost‐efficient healthcare strategies and squeezed resources for patient care have made the phenomenon more widespread and noticeable.

## 2 BACKGROUND

Recent works in literature have recognised both individual and collaborative forms of workaround and have classified it into three main categories relating to technology, work restraints and policies/rules/regulations (Debono et al., 2013). Based on the circumstances and situations in which workarounds are implemented, the phenomena can lead to differing or even opposite effects in the clinical practice: from improving the efficiency of workflow to increasing patient safety or even to establishing unstable or unreliable work procedures that negatively influence the safety, effectiveness and efficiency of care (Blijleven et al., 2017). In fact, with a workaround, nurses, for instance, may bypass essential blocks defined by the organisation to ensure safety to patients and employees, eventually harming them. A workaround may help overcome immediate obstacles, for example, caused by a dysfunctional technological work system that delays quick intervention (Heron & Bruk‐Lee, 2020) but is not effective long‐term, as it leaves the underlying problem unsolved.

However, from a regulatory aspect, the workaround is basically a non‐compliance, a deviation from and a violation of an organisation's defined procedures, regulations, standards or rules that can contribute to adverse events (Adachi et al., 2016; Gorini & Pravettoni, 2013). Evidence from meta‐analyses shows workaround being promoted by both organisational factors (staffing levels, skill mix, workload, poor leadership, insufficient resources and support) and work process factors (i.e. uncompleted documentation, insufficient tools, and equipment) (Heron & Bruk‐Lee, 2020). Therefore, nurse leaders need to be aware of the phenomenon, measure its extent within the organisation, understand the underlying causes and reconcile formal procedures with the highest efficiency level in clinical practice.

Despite the vast literature on workarounds, so far, few works have explored experience, results or lessons learned of the workarounds from a nurse's perspective.

## 3 METHOD

### 3.1 Aim

This study aimed at exploring the phenomenon from the nurses’ point of view and based on their experience with activities carried forth non‐compliantly to procedures, protocols and rules defined by the Institution during their clinical activity.

### 3.2 Design

The study followed Colaizzi’s (1978) phenomenological descriptive method, which is most suitable for gaining insight into subjective meaning‐making about personal experiences and the participants’ motivations and actions (Streubert & Rinaldi Carpenter, 2011).

The purpose of phenomenological research is to understand the essence of social phenomena from the perspective of those who perceive them (Creswell, 2007). Based on the research question, the phenomena to be described and generated as knowledge by the researchers in our enquiry was the experience of workarounds perceived by hospital nurses. We adopted a specific approach to phenomenological investigations, descriptive phenomenology (Giorgi, 2012), rather than the interpretative approach (Smith et al., 2009). The latter relies on a hermeneutic perspective on data, requiring researchers to make sense of the participants trying to make sense of what is happening to them (double hermeneutic). Contrariwise, researchers conducting descriptive phenomenology try to bracket their interpretation of phenomena to give room to the essence of the experience as revealed by participants’ voices, retaining them without abstracting their viewpoint out through analysis. In other words, employing descriptive phenomenology means ‘to capture as closely as possible how the phenomenon is experienced within the context in which the experience takes place’ (Giorgi & Giorgi, 2003, p. 27). For reaching such closeness, descriptive phenomenologists employ in‐depth interviewing (Patton, 1990). Colaizzi (1978) developed a descriptive method from Giorgi's indications, primarily recognised in healthcare research. The data analysis plan used in this study was Colaizzi’s (1978) descriptive phenomenological method. It foresees the ‘transcription of the description provided by each’ and then ‘extracting significant statements’ for ‘creating formulated meanings’ and ‘aggregating them into theme clusters’, based on which researchers then reach ‘an exhaustive description’ and ‘the fundamental structure of the phenomenon’ (Colaizzi, 1978). The last step of Colaizzi's method includes a findings’ validation from participants who can verify results with their experiences.

### 3.3 Setting

The commissioners of this investigation requested the researchers to conduct the study in a specific hospital in Canton Tessin (Switzerland). Given this indication, the authors made a list of hospital departments. They then selected two unique settings comparable in terms of the numbers of employed professionals, the complexity of care, staffing and skill mix. The inclusion criteria for participants were nurses with at least 2 years of professional experience, having worked in the hospital for at least 2 years. The researchers decided on criteria, hypothesising that 2 years of experience in the role and the department was sufficient to understand the rules, setting and organisational climate. At the beginning of the study, we planned to interview around 12 nurses; the sample, however, was expanded to include interviews with four more nurses.

The principal investigator (first author) acted as gatekeeper and provided information about the study and consent. Participants were first contacted via e‐mail asking their willingness to be involved in the research and then a phone call to get to know the researcher and schedule the interview. All the contacted participants accepted the invitation.

### 3.4 Data collection

Data were collected from October to November 2019 with in‐depth interviews, consisting of an opening question and a collection of prompts to be asked in a flexible order to investigate further aspects emerging during the interview (Sasso et al., 2015). The same researcher (first author), an expert in qualitative research and nursing, conducted the interviews in the participants’ workplace because it was convenient and could ensure confidentiality. In addition, demographic data were collected. The interviews ranged from 45’ to 90’, were digitally audio‐recorded with the participants’ consensus.

### 3.5 Ethical considerations

The cantonal Ethics Committee was approached by the researchers, who were informed that, after evaluation of the research project, formal approval by the Ethics Committee was not needed according to the Human Research Act (The Federal Assembly of the Swiss Confederation, 2011).

The researcher, however, adopted specific participants’ protection measures. Participants were adequately informed about the aim and participation procedures. They were also informed about confidentiality: they were reassured that the data collection method would not result in personal identification or harm. Participation in interviews was voluntary, with the right to withdraw without consequences. The researcher gathered written consent before audio recording and audio‐registered the consent soon after. It was also clearly stated that all and any information obtained as a consequence of this study would be guaranteed and protected as indicated under the Federal Law on Privacy and, as such, could not possibly be traced.

### 3.6 Data analysis

The interviewer verbatim transcribed interviews. Data analysis was done simultaneously with the data collection by the authors independently and followed, for each transcript, three phases:

‐ Extraction of significant statements after the interviews were transcribed.
‐ Creation of formulated meanings.
‐ Aggregation of formulated meanings into themes (Colaizzi, 1978).

The researchers met afterwards and shared the provisional findings to reach an intercoder agreement. Themes and sub‐themes were generated across all the datasets, based on consensus among researchers. The first author organised an event of results sharing with all participants 6 months after data collection. All 16 participants attended and had the opportunity to intervene and bring back their suggestions and ideas. No managers or research commissioners were present, so participants were likely to feel comfortable. What emerged from the discussion confirmed the overall findings the researchers had reached and was considered for this final version of the results. The process employed NVivo 12 software.

### 3.7 Bracketing and rigour

To reduce social desirability bias, the interview was administered by a researcher independent of the hospital (Krumpal, 2013). Given the topic's sensitivity, we constructed a participant relationship based on listening and a non‐judgmental approach. We managed to bracket preconceptions coming from the first author's professional background (she is a university professor with expertise in nursing practice) and previous knowledge of the phenomenon (Giorgi, 2012) by posing open‐ended questions during the interview to allow participants to share their points of view on their experiences freely. Besides, the second author (with a background in education and an expert in qualitative methods) served as a non‐nursing researcher to avoid data analysis confirming likely preconceived expectations. The two authors analysed data, and differences in interpretations were solved through discussion. The analysis credibility and originality were obtained by gathering rich, in‐depth data from interviews, transcribing verbatim and using the participants’ own words as much as possible (Finlay, 2002). An external audit was conducted by an expert researcher, who checked the interview transcripts and analysis to ensure rigour.Finally, a member‐check of synthesized analyzed data was carried out with the aim of exploring whether results had resonance with the participants’ experience (Birt et al.,^2016^).

## 4 RESULTS

The study involved a total of 16 participants, whose characteristics are described in Table 1.

**TABLE 1:** Participants’ characteristics

N°	Age range	Work experience (in years, range)	Work experience in the medical ward (in years)
1	41–45	11–15	6
2	51–56	21–25	13
3	26–30	1–5	2
4	46–50	21–25	25
5	26–30	1–5	3
6	26–30	6–10	6
7	26–30	1–5	4
8	26–30	1–5	4
9	46–50	16–20	18
10	26–30	1–5	3
11	31–35	6–10	8
12	41–45	1–5	4
13	41–45	1–5	3
14	31–35	6–10	10
15	46–50	26–30	16
16	31–35	1–5	2.5

At the end of data analysis, we obtained 17 sub‐themes that fit into in four broader themes (Table 2): (i) living the profession in saved times; (ii) perceiving contingencies as a guide to action; (iii) sense of personal responsibility; and (iv) emotional aspects.

**TABLE 2:** Major themes and sub‐themes

Themes	Sub‐themes
Living the profession in saved times	Functionality as saved time
	Experiencing protocols as acts I can derogate from
	Reasoning asynchronously from reality
	Selecting priorities
Perceiving contingencies as a guide to action	Necessity guides the action
	Opportunities
	The primacy of experience
	Lesson learned
	Relevance of consequences
Sense of personal responsibility	Professional competencies legitimise decisions
	Responsibility—weight and sharing
	Individual responsibility
	Value of one's own conscience
	Deontological and professional ethical aspects
Emotional aspects	Living the feelings
	Perceiving the separation between private and public time
	Relationships with superiors

These themes were then interpreted as a whole and used to define the following overarching statement.

Based on strict protocols that need to be observed, professionals interpreted their profession in time saved from the strict abiding to such protocols. They lived the protocol accordingly as a set of acts they could deviate from (Living the profession in saved times) during their clinical practice. The reasons supporting their turn towards non‐compliant practices were triggered by the multiple situations experienced (interiorised), hence by criteria dictated by the contingencies. They believe that the protocol was ‘non‐specific’ (i.e. general guidance); therefore, their actions, in reality, were guided by the acts that they, as professionals, considered the most appropriate (*Perceiving contingencies as a guide to action*). For them, living and experiencing workaround within the defined framework (protocol) was not neutral from an ethical point of view. Instead, it impacted their sense of responsibility and their experience linked to acts of conscience (*Sense of personal responsibility*). Lastly, they considered this way of perceiving protocols, rules and consequential acting, as non‐neutral from an ethical and conscientious point of view and an emotional point of view (*Emotional aspects*).

### 4.1 Living the profession in saved times

Being forced to follow very often strict rules and protocols during clinical activities, professionals perceived that they could live their work only in the ‘times’ they were able to ‘cut out’, ‘save’ from not following all the steps imposed by the procedures. Within these times, they felt they could accomplish the essence of what they defined as ‘their profession’.

#### 4.1.1 Functionality as saved time

According to the participants, the procedures were not functional to manage the clinical activities they had to carry out. Within the times in which these needed to be carried out, so many of them expressed the non‐feasibility of the procedures defined ‘imposed’ onto them.

There have been instances in which I hadn't followed the procedures because … sometimes they are not very feasible and not very functional for daily work (…) they are not quite applicable. Not applicable, because we have to respect the times, because we have to consider the unexpected, because there is a whole series of factors that sometimes … make it difficult for you to follow the theory. (N 1)

#### 4.1.2 Experiencing protocols as acts I can derogate from

Within the defined procedures, the participants identified several actions and clinical activities that, in their opinion, were of minor importance and could be ‘skipped’ or avoided.

Sometimes I don't follow the protocol, in the sense that uh thirty seconds seems nothing, but it's a lot of time … so sometimes, I decide not to respect it because of a timing issue. (N 5)

#### 4.1.3 Reasoning asynchronously from reality

The professionals found themselves rushing through tasks without time to think about what they had to do next. They tried to make up for this shortcoming once their shift was over, rethinking and reasoning about the work carried out: some did it on the work‐home route while others did the rest time.

Then you think again there: but did you remember to put gloves there? To disinfect your hands after … after contact with this patient? Yes, think about it, and go over everything again. (N 13)

Then you get in your car, an hour's drive home, and you think, you think, but did I do this? Did I do that? Did I write this down? I think a lot, too much sometimes, maybe. (…) Looking back, looking back … the work shift, there were moments I couldn't remember if I did that specific thing or not. And so … (N 3).

#### 4.1.4 Selecting priorities

As described by participants, requirements by protocols, procedures and rules defined by the Institution often exceeded the capability of limited staff available to perform them. Professionals were overwhelmed by tasks and responsibilities and were aware of their limits in accomplishing them within the time available. For some individuals, such failure in carrying out their duty lingered within as a sense of frustration, while for others, it represented the drive to increasing self‐efficiency. In fact, with the lack of official guidance on ‘making time’, professionals tended to become creative, finding short‐cuts or reassigning priorities based on their perception of urgency and guided by what was best for the patients.

They select the priority (…) in front of the main priority. Maybe the other things have been subordinated later. But in principle, you always try to … just one way to … (N 11)

I would never let it slip my mind between one dressing and another, between one sample and another, between one pose of a venflon and another, between … invasive measures, biological fluids, it would never escape me. (N 8)

### 4.2 Perceiving contingencies as a guide to action

In fact, within this theme and in its sub‐themes, all professionals described these choices as being guided by needs of the moment that required a quick response or contingency.

#### 4.2.1 Necessity guides the action

In the event of quick decision‐making, many participants described their actions as guided by the moment's needs.

You can, but … many times … on a typical day, if something different happens, if someone is sick, if he falls on the floor … in theory, you should disinfect your hands before touching him … but someone is sick (…) uh, in an emergency the first thing you think of is the person and not the procedure! (N 2)

#### 4.2.2 Opportunities

Another way to guide the identified action was that of appropriateness: what was considered appropriate or not.

Hmm, otherwise, until that moment, I remember that we did our evaluation, we did some tests, asked the patient, and then … if we thought it was appropriate … we raised it, that's it. (N 12)

#### 4.2.3 The primacy of experience

Overall, reactions and responses to patients’ needs by the professionals we interviewed were driven by experience gained over the years that guides people to respond to needs. Participants were confident that experience enabled them able to make the most appropriate decisions.

It's not so much a decision, but it's more of automatism that I don't have, in a sense … we don't … I mean, at least, for me … but I think a little bit for everyone, it wasn't really so imperative when I started working here and when I went to school … and so certain brain mechanisms lead you to do this. (N 6).

Yes, no no, it's normal that with experience, you understand that maybe it's not appropriate to do it. (N 14)

#### 4.2.4 Lesson learned

Participants replied with different approaches; the most important was learning from the mistakes and realising that their mistakes were a life lesson for them.

That is, for me, this is something that I learned. I was not like that when I had just started, just graduated, it seemed to me to … with hindsight, I was taking a bit lightly this issue of … security. I seemed to say ah, but yes, I’m capable … actually then, with experience, seeing and making mistakes, I realised how important this side is anyway and that it is not good to be so ‘approximate.’ (N 7).

#### 4.2.5 Relevance of consequences

Based on the consequences they thought may occur or that they had actually experienced in the past, they decided how to act, whether or not to follow all the steps, and/or how to orient themselves.

We made a first evaluation of the consequences … we thought there were no relevant consequences and (…) I doubt that there could be any serious consequences for staff not disinfecting their hands every time they touch a tray, serve it, and put it on the patient. (N 10)

### 4.3 Sense of personal responsibility

Professional responsibility was sometimes experienced by participants as a burden, a boulder on their shoulders. Many of them told how they felt relieved of this burden when they could share it with their colleagues when the responsibility was ‘spread’.

#### 4.3.1 Professional competencies legitimise decisions

Several participants stressed the weight of their professional experience and specialistic skills as a criterion for their clinical decision‐making.

What I mean is that I first assess the patient is – I clearly know the treatment I have to administer him– to provide a certain comfort, to make him feel better. I obviously adapt it to the patient, to his situation: so, it's true, there are standardised protocols, but you are not always able to implement them. (N 8)

#### 4.3.2 Responsibility—weight and sharing

Another aspect that nurses mentioned was the need for approval for their undertakings. They were aware of acting non‐compliantly to procedures and taking on added responsibilities. When there were colleagues on shift with whom to share decisions, the burden of responsibility was lightened. Responsibility was perceived as being distributed, and by sharing and discussing opinions, care teams were created and consolidated.

In the meantime, we have … put him back to bed. This has been shared with others. (…) If there are other nurses obviously … the responsibility is shared, in the sense that there is an exchange of opinions, if then … there is a common opinion … it helps to act. (N 16)

#### 4.3.3 Individual responsibility

On the other hand, participants evidenced the lack of approval or support to their decisions when they were alone on shift or, at least, they felt that they were alone, and the feeling of total responsibility for their actions and clinical decisions. During the interviews, they described the term ‘loneliness’ as being alone during the work shift or linked with the composition of the staff mix, like when the colleagues present did not have the same level of training and, consequently, responsibility.

And then, especially in those cases where, when maybe you were alone, with such an assistant, you felt much more responsible, because any way you had no way to … to exchange or share, an opinion. (N 14)

#### 4.3.4 Value of one's own conscience

Regardless of the moments of sharing, peer discussion and shared decisions, participants placed great value on following their conscience to act and making decisions in clinical practice.

As far as I am concerned … I have to answer to my conscience first. (N 1)

#### 4.3.5 Deontological and professional ethical aspects

The participants defined the ethical and deontological values of the profession as an essential guide for their actions.

I always use the technique not to do to others what I would not like to be done to me, so … not only I want to protect the person because it is ethically correct and because it is from a personal and spiritual point of view. (N 15)

### 4.4 Emotional aspects

Participants told an experience with feelings of guilt, fear, fear and anxiety that they were often unable to circumscribe to the context of work and working time but that invaded private time. These emotional aspects, it was reported, could be exacerbated or resolved according to the types of relationships that professionals have with their superiors.

#### 4.4.1 Living the feelings

Non‐compliance to protocols and personal initiatives generated mixed feelings among participants. To some, the burden of added responsibility lingered over time and, in some cases, remained vivid even through the years.

I always feel a little guilty. Always! That's because … That is, I feel a bit at fault … even if I give a certain weight, you know, to everything, I give a certain weight. I always feel at fault because if I know, I should do it in a way … I always have it. (…) I was devastated. I mean, it was something that had never ever happened to me, and instead … (…) And so it was … it was a defeat, you know. Then you get over it … (N 4).

#### 4.4.2 Perceiving the separation between private and public time

The participants described how concerns and emotions were so significant and so substantial that they could not restrict them in the workplace and that they often carried over into their private life.

I went home. I was also a little sorry, and I said ‘poor thing.’ I think … even when at I am home, I say … who knows how tonight will go, will he make it … and I stay a bit … because when I leave the situation unresolved, it's a bit a thought, I hope that … (…) I’m thinking about him until one in the morning, I wake up and say, let's hope that… that he's better … (N 9).

#### 4.4.3 Relationships with superiors

Talking and confronting the superior could reassure professionals. Many participants said they were looking for this exchange because they felt welcomed and understood.

I never had any problem with him, he always listened to me, I could always tell him ‘look, this is what happened’ if I was wrong, tell me if I have to make some report, there is no problem (N 7).

## 5 DISCUSSION

The present study investigated the circumstances and triggers for workarounds in clinical practice through the eyes and experience of nursing staff members. Findings have evidenced workaround as a symptom of unmet professional needs and lack of time or tools to complete their tasks by the book.

The results of this research do not stress the presence of organisational factors and work process factors as creating the condition to use workaround but are more linked to the professional needs; among these, the most important described by participants is to have time to express the fundamental meaning of being a nurse (*Living the profession in saved times*). If nurses use a workaround, they can earn time to dedicate to stringent time restraints, emergencies, solving workflow barriers, avoiding interruptions, enhancing communication, managing heavy workloads related to the type of patient (Alper et al., 2012; Flanagan et al., 2013) and—as often explained in the participants’ quotes—to caring for their patients, maintaining a good relationship with them and their relatives, and establishing empathy.

As noted by Halbesleben et al. (2008), one of the motivations that induce the healthcare professionals to use workarounds is to seek to consider the organisational requests and, at the same time, they need to provide patient‐centred care. The research in this field shows that healthcare workers improvise or use the workaround to provide patient‐centred care guaranteeing efficiency and cost‐effectiveness (Lalley & Malloch, 2010).

From our analysis, we can state that doing workarounds in clinical practice, or even rule violations, involves ethical dilemmas, particularly those related to organisational constraints and patients’ dignity, as described by Rainer and colleagues (Rainer et al., 2018). In their integrative review, organisational constraints entail staffing shortage as the cause for nurses to determine what they could omit (rationing care) (Ball et al., 2014).

Similarly, our participants found short‐cuts or reassigned priorities based on their perception of urgency in the context of limited staff available (Cooper et al., 2004). However, in our study, findings evidenced how organisation‐related ethical dilemmas may be determined by staff shortage and how protocols, procedures and rules had been designed.

Besides, workaround and violations were part of an ethical dilemma referrable to patients’ dignity and privacy, identified as a cause of dilemmas elsewhere (Rainer et al., 2018). From our study, participants were guided by what was best for the patients, according to contingencies and relevance of expected consequences. The decisions of rule violation involved their responsibility. So, ethical awareness may emerge along with the very acts of workaround having moral consequences (Milliken & Grace, 2017), as noted by our participants.

In this context, care ethics as a theoretical framework can help interpret such violations in healthcare. Care ethics is a theoretical framework including the belief systems of healthcare professionals (De Panfilis et al., 2019) capable of explaining workaround into a broader picture (not only in a legalistic way).

In the international debate, ample space is being given to ‘being a good nurse and doing the right thing’ and the link that this has with the world inside and outside the job (Catlett & Lovan, 2011; Debono et al., 2013); in this study, this link is underlined related to *Emotional aspects* where participants reported how they live feelings, the difficulties in separating private and public time and the heaviness of living the sense of guilt, fear and anxiety.

Professional and organisational standards, procedures, pathways and guidelines, define the way that nurses ‘should’ follow, consistently with what they are taught during their education; yet nurses describe the ‘real world’ as complex, and their everyday experience as the perceived need to delivering care in a complicate adaptive system where the workaround is necessary (Debono et al., 2012)—above all when they perceive that they are guided by the contingencies (*Perceiving contingencies as a guide to action*).

An organisational culture where nurses can share responsibilities, have support from the leaders, be empowered and be involved in the changes are essential aspects that emerge from the reflections on the *sense of personal responsibility* and contribute to reducing workaround (Adachi et al., 2016).

Defining the effect of workarounds on performance is not straightforward because they can have different effects on health outcomes, costs and quality of service. Some literature underlines that workarounds can contribute to errors (Koppel et al., 2008; Tucker, 2016), safety and poor patient outcomes (Tucker et al., 2020). They can also decrease performance and organisational learning (Debono et al., 2012; Mazur et al., 2009; Naveh & Marcus, 2005; Spear & Schmidhofer, 2005).

## 6 CONCLUSIONS

This study aimed to investigate the experience of nurses when they do not follow protocols defined by an Institution during their activity. The chosen method, phenomenology, allowed us to describe the phenomenon and discover aspects rooted in the people's experiences included in the study. We could deepen the meaning of their experience by seeing it from their perspective. An interesting development of this research would be setting the groundwork for joint protocol design involving both management and clinical nurses, eventually leading to a reduced need for a workaround in future.

### 6.1 Strengths and limitations

Given the setting and the location of the study, findings do not allow for broad generalisation but yet are readily transferable in other contexts. They provide insights into feelings, meanings and lived experiences occurring whenever protocols and rules must be obeyed in clinical settings. The four themes can become the basis for further study and for considering a possible application to similar clinical contexts. The choice to interview nurses alone can be changed to open interviews with other professionals involved in clinical teams.

## 7 RELEVANCE TO CLINICAL PRACTICE

The results of this study can help clinical nursing leadership acknowledge workaround (also in its ‘lived’ meaning) and address its triggers. Nursing managers should comprehend workarounds are not done as violations and do not necessarily implicate missed care. Missed care involves leaving care undone. On the contrary, workarounds were described by our participants as strategies for being close to patients and fulfilling their sense of ‘being a nurse’.

Acknowledging how nurses perceive this phenomenon is pivotal for clinical nursing leaders who could use the information from the study to support nurses and create conditions that improve a way to work in clinical practice without a workaround. For example, they could schedule periodic meetings to collect and discuss workaround and causes and attempt to reconcile them by introducing newly revised procedures, raising awareness among professionals about the problem and the risks it entails, and identifying some possible solutions at an organisational level.

Increased knowledge in these fields contributes to creating safer organisations, improve patient outcomes, professionals’ satisfaction and empower the nursing team.

## Supporting information

Supplementary Material

## Data Availability

Data available on request from the authors.
