# Supplementary material for: Nurses’ perspectives on workarounds in clinical practice: A phenomenological analysis
Source: J Clin Nurs. 2021 Nov 9;31(19-20):2850–9. doi: 10.1111/jocn.16110 (PMC9539462; doi:10.1111/jocn.16110)
Supplement: Supplementary file 1 — Supplementary Material [file JOCN-31-2850-s001.docx]

Coreq

| **Domain 1: Research team and reflexivity** | Item  No. | Description | Reported on  Page No. |
| --- | --- | --- | --- |
| *Personal Characteristics* |  |  |  |
| Interviewer/facilitator | 1 | Which author/s conducted the interview or focus group? | 6 |
| Credentials | 2 | What were the researcher’s credentials?  E.g. PhD, MD | Title page + 6 |
| Occupation | 3 | What was their occupation at the time of the study? | Title page |
| Gender | 4 | Was the researcher male or female? | Title page + 6 |
| Experience and training | 5 | What experience or training did the  researcher have? | 6 |
| *Relationship with participants* |  |  |  |
| Relationship established | 6 | Was a relationship established prior to study commencement? | 6 |
| Participant knowledge of the interviewer | 7 | What did the participants know about the researcher? e.g. personal goals, reasons  for doing the research | 6 |
| Interviewer characteristics | 8 | reasons and interests in the research topic | 6-7 |
| **Domain 2: study design** |  |  |  |
| *Theoretical framework* |  |  |  |
| Methodological orientation and Theory | 9 | What methodological orientation was stated to underpin the study? e.g. grounded theory, discourse analysis, ethnography, phenomenology, content  analysis | 5 |
| *Participant selection* |  |  |  |
| Sampling | 10 | How were participants selected? e.g. purposive, convenience, consecutive,  snowball | 5 |
| Method of approach | 11 | How were participants approached? e.g. face-to-face, telephone, mail, email | 6 |
| Sample size | 12 | How many participants were in the study? | 6 |
| Non-participation? | 13 | How many people refused to participate  or dropped out? Reasons? | 6 |
| *Setting* |  |  |  |
| Setting of data collection | 14 | Where was the data collected? e.g. home, clinic, workplace | 6 |
| Presence of non-participants | 15 | Was anyone else present besides the participants and researchers? | 6 |
| Description of sample | 16 | What are the important characteristics of  the sample? e.g. demographic data, date | 8, table 1 |
| *Data collection* |  |  |  |
| Interview guide | 17 | Were questions, prompts, guides provided by the authors? Was it pilot tested? | Table 1 |
| 18. Repeat interviews | 18 | Were repeat interviews carried out? If  yes, how many? | NA |
| Audio/visual recording | 19 | Did the research use audio or visual recording to collect the data? | 6 |
| Field notes | 20 | Were field notes made during and/or  after the interview or focus group? | 6 |

| Duration | 21 | What was the duration of the interviews  or focus group? | 6 |
| --- | --- | --- | --- |
| Data saturation | 22 | Was data saturation discussed? | NA |
| Transcripts returned | 23 | Were transcripts returned to participants  for comment and/or correction? | 7 |
| **Domain 3: analysis and findings** |  |  |  |
| *Data analysis* |  |  |  |
| Number of data coders | 24 | How many data coders coded the data? | 7 |
| Description of the coding tree | 25 | Did authors provide a description of the  coding tree? | 8 |
| Derivation of themes | 26 | Were themes identified in advance or  derived from the data? | 8 |
| Software | 27 | What software, if applicable, was used to  manage the data? | 7 |
| Participant checking | 28 | Did participants provide feedback on the  findings? | 7 |
| *Reporting* |  |  |  |
| Quotations presented | 29 | Were participant quotations presented to illustrate the themes / findings? Was each  quotation identified? e.g. participant number | 8-18 |
| Data and findings consistent | 30 | Was there consistency between the data  presented and the findings? | 8-18 |
| Clarity of major themes? | 31 | Were major themes clearly presented in  the findings? | 8-18 |
| 32. Clarity of minor themes | 32 | Is there a description of diverse cases or  discussion of minor themes? | 8-18 |

Adapted for word

Developed from: Tong A, Sainsbury P, Craig J. Consolidated criteria for reporting qualitative research (COREQ): a 32-item checklist for interviews and focus groups. International Journal for Quality in Health Care. 2007. Volume 19, Number 6: pp. 349 – 357

Once you have completed this checklist, please save a copy and upload it as part of your submission. DO NOT include this checklist as part of the main manuscript document. It must be uploaded as a separate file.
